# Cerebral Venous Sinus Thrombosis Following Pneumococcal Meningitis: A Rare but Serious Complication

**DOI:** 10.7759/cureus.111348

**Published:** 2026-06-23

**Authors:** Mohammed Mosharraf Hossain Khandaker, Zhiyu Loh, Yasir Mehmood, Syed Abubacker, Syed Jafri

**Affiliations:** 1 Acute Medicine, Kettering General Hospital NHS Trust, Kettering, GBR; 2 Internal Medicine, University Hospitals of Leicester NHS Trust, Leicester, GBR

**Keywords:** anticoagulation, complication of meningitis, meningitis, pneumococcal meningitis, venous sinus thrombosis

## Abstract

Cerebral venous sinus thrombosis (CVST) is a rare but important complication of bacterial meningitis and is most commonly associated with *Streptococcus pneumoniae*. We report the case of a 60-year-old man who developed extensive CVST following the completion of a 14-day course of intravenous ceftriaxone for pneumococcal meningitis. Despite biochemical resolution of infection and absence of focal neurological deficits, persistent headache and meningism prompted further neuroimaging, which confirmed the diagnosis. He was treated with anticoagulation, resulting in subsequent radiological resolution of thrombosis, although headaches persisted following recanalisation.

## Introduction

Bacterial meningitis remains a serious and potentially life-threatening infection associated with significant morbidity and mortality despite advances in antimicrobial therapy [1]. *Streptococcus pneumoniae* is the most common causative organism in adults and is frequently associated with severe disease and neurological complications [2].

Cerebral venous sinus thrombosis (CVST) is a rare but recognised complication of bacterial meningitis. A large Dutch nationwide prospective cohort of 2220 patients reported an incidence of CVST complicating meningitis of approximately 1%, with *Streptococcus pneumoniae* identified as the most common causative organism [3]. CVST is often identified at or near presentation, particularly in patients with more severe disease or concurrent sinus or ear infections [3].

The clinical presentation of CVST in this context can be subtle and overlap with features of the primary infection, notably headache and meningism [2,4]. Persistence of these symptoms, or the development of new neurological deficits despite appropriate antimicrobial therapy, should prompt consideration of CVST.

We present a case of extensive cerebral venous sinus thrombosis involving the transverse and sigmoid sinuses, as well as the internal jugular vein, occurring shortly after treatment for pneumococcal meningitis. This case highlights the importance of considering CVST in patients with persistent symptoms despite biochemical and clinical improvement and underscores the role of early neuroimaging in guiding diagnosis and management.

## Case presentation

A 60-year-old male with a background of asthma and anxiety re-presented to the Same Day Emergency Care (SDEC) unit with ongoing headache, neck pain, and photophobia 1 day after completing a 14-day course of intravenous ceftriaxone for confirmed pneumococcal meningitis.

The initial diagnosis of meningitis had been established during a prior admission following lumbar puncture (LP), which revealed turbid cerebrospinal fluid (CSF) with polymorphic pleocytosis and increased protein (Table 1). CSF polymerase chain reaction (PCR) was positive for *Streptococcus pneumoniae*, confirming the diagnosis.

**Table 1:** Cerebrospinal fluid results

Cerebrospinal fluid	Results	Reference range
White cell count	2020 x 10^6^/L	0-5 cells x 10^6^/L
Protein	4.72 g/L	0.18 - 0.58 g/L
Glucose	2.5 mmol/L	2.8 - 4.4 mmol/L

Despite completion of appropriate antimicrobial therapy, the patient reported only partial improvement in symptoms, with persistent headache and meningism since the onset of illness. There was no history of new fever, seizures, focal neurological deficits, or altered consciousness. There was also no history suggestive of other prothrombotic risk factors such as recent surgery, malignancy or known thrombophilia.

On examination, the patient was afebrile with stable observations. Neurological examination demonstrated no focal deficits. Cranial nerve examination was normal. There was no rash. Fundoscopy was not performed at this presentation. However, Kernig’s sign remained positive, consistent with ongoing meningeal irritation.

Blood investigations at this stage (Table 2) were within normal limits, with a normal white cell count, C-reactive protein, renal function tests and liver function tests. This represented a marked improvement compared to his initial presentation, where inflammatory markers were significantly elevated.

**Table 2:** Blood investigations GFR=Glomerular Filtration Rate, ALT=Alanine Aminotransferase, ALP=Alkaline Phosphatase

Blood results	Initial presentation	Second presentation	Reference range
White cell count	21.8 x 10^9^/L	5.8 x 10^9^/L	4.0 - 11.0 x 10^9^/L
Haemoglobin	139 g/L	146 g/L	130 - 180 g/L
Platelets	211 x 10^9^/L	439 x 10^9^/L	150 - 450 x 10^9^/L
Neutrophils	20.4 x 10^9^/L	3.6 x 10^9^/L	2.0 - 7.5 x 10^9^/L
C-Reactive Protein	262 mg/L	<5 mg/L	<5 mg/L
Sodium	136 mmol/L	141 mmol/L	133 - 146 mmol/L
Potassium	4.1 mmol/L	4.8 mmol/L	3.5 - 5.3 mmol/L
Urea	4.5 mmol/L	4.8 mmol/L	2.1 - 7.1 mmol/L
Creatinine	77 µmol/L	75 µmol/L	59 - 104 µmol/L
Estimated GFR	>90 ml/min/1.73m^2^	>90 ml/min/1.73m^2^	>90 ml/min/1.73m^2^
ALT	14 IU/L	35 IU/L	<41 IU/L
ALP	85 U/L	80 U/L	30 - 130 U/L

Given the persistence of symptoms despite adequate treatment, further investigation was pursued. A repeat lumbar puncture was recommended to assess for ongoing inflammation or alternative pathology. However, the patient declined this procedure. Magnetic resonance imaging (MRI) of the brain (Figure 1) was performed, which demonstrated loss of normal flow void within the right transverse sinus, right sigmoid sinus and visualised right proximal internal jugular vein, raising suspicion for CVST.

**Figure 1:**
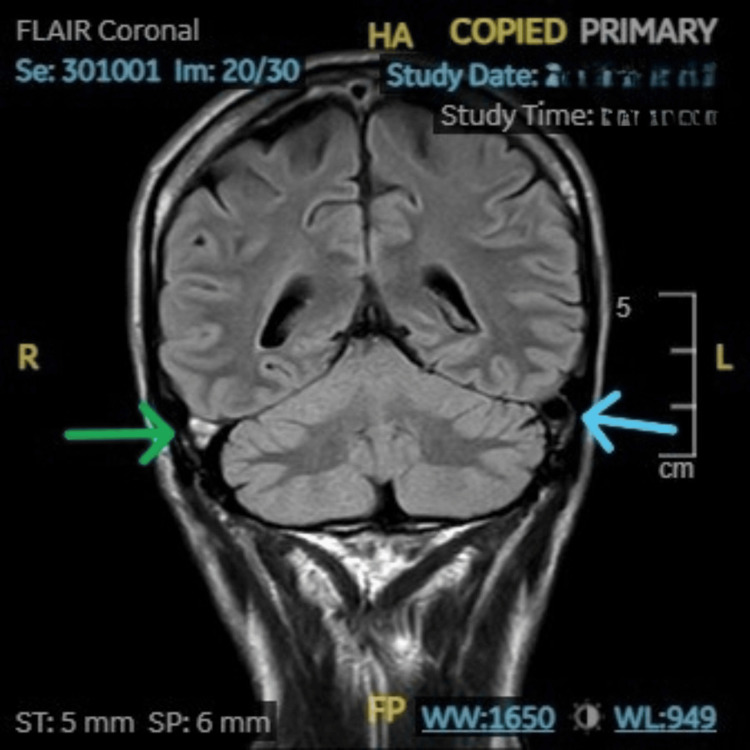
MRI brain raising suspicion of CVST Green arrow – High signal of thrombosed blood in the right sigmoid sinus. Blue arrow – Signal void in the patent left sigmoid sinus. CVST=Cerebral Venous Sinus Thrombosis

A subsequent magnetic resonance venogram (MRV) (Figure 2) confirmed extensive thrombosis involving the right transverse sinus, sigmoid sinus, and proximal internal jugular vein.

**Figure 2:**
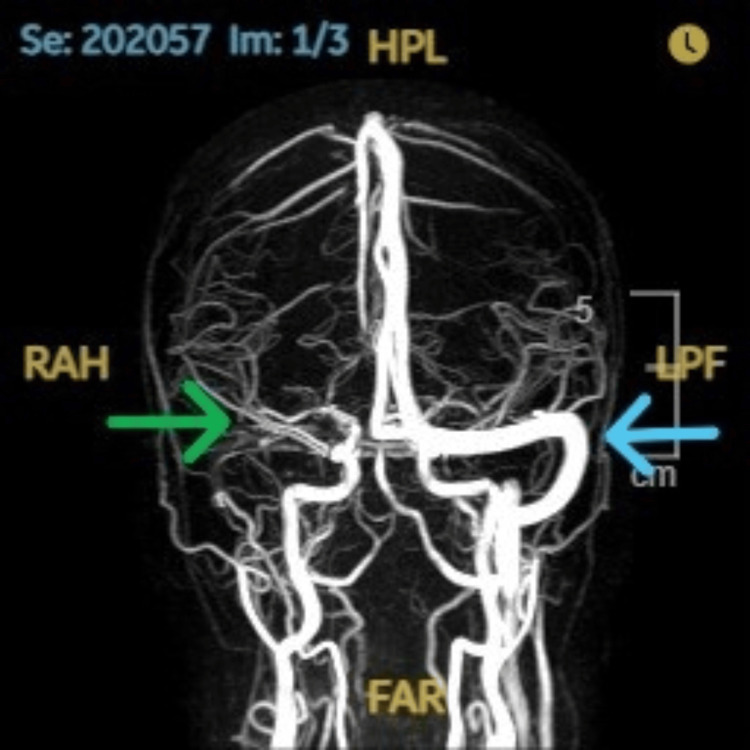
MR venogram confirming extensive CVST Green arrow – No flow in the thrombosed right transverse sinus. Blue arrow – Patent flow in the left transverse sinus. CVST=Cerebral Venous Sinus Thrombosis

The patient was commenced on therapeutic-dose enoxaparin following a multidisciplinary discussion with Haematology. Further investigations for underlying prothrombotic states, such as antiphospholipid syndrome, polycythaemia vera and paroxysmal nocturnal haemoglobinuria, were negative. He was then switched to apixaban after the initial phase. He developed generalised pruritus with both apixaban and rivaroxaban and was therefore switched back to therapeutic-dose enoxaparin. He completed approximately four months of anticoagulation before deciding to discontinue treatment because of medication-related side effects.

At five months after the diagnosis of the CVST, a CT venogram (Figure 3) demonstrated no evidence of residual thrombosis.

**Figure 3:**
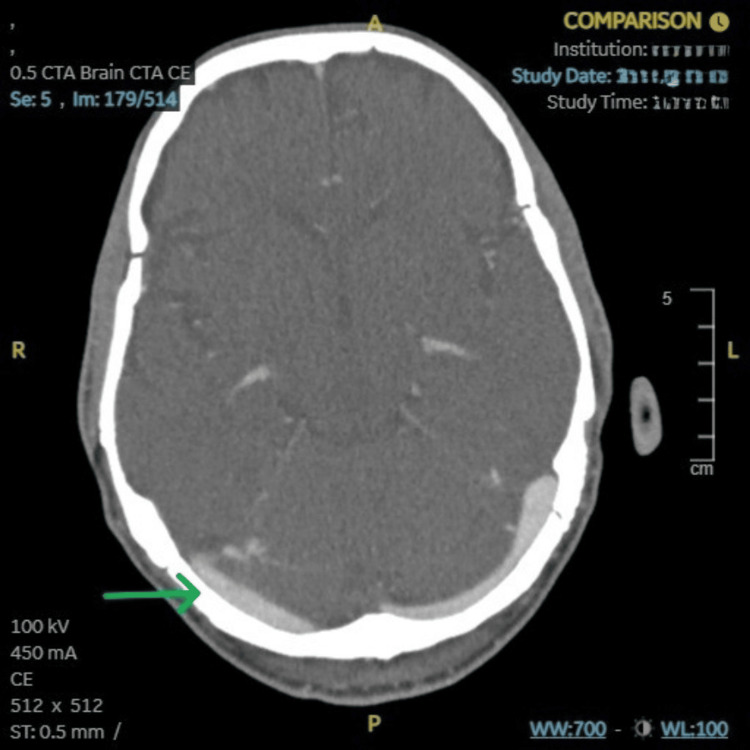
CT venogram five months after diagnosis Arrow – Patent recanalised right transverse sinus post-treatment.

He was reviewed by Neurology, which suggested that his persistent headaches at this point had features of migraine and recommended migraine prophylaxis.

He re-presented to the SDEC one year after the initial diagnosis with persistent headaches. Repeat CT venogram showed no evidence of recurrent CVST. He was discharged with appropriate safety netting advice.

## Discussion

CVST is a recognised but uncommon complication of bacterial meningitis. The underlying pathophysiology is multifactorial. Infection may contribute to thrombosis through several mechanisms, including vasculitis predominantly affecting vessels at the base of the brain, immune-mediated parainfectious processes leading to vasospasm or thrombosis and a hypercoagulable state driven by endothelial dysfunction and activation of inflammatory and procoagulant pathways [5]. In addition, direct extension of infection from adjacent structures, particularly in the presence of concurrent mastoiditis, may further contribute to venous sinus involvement [3]. Collectively, these processes predispose to thrombus formation within the cerebral venous system.

The reported incidence of CVST in bacterial meningitis is low but likely underrecognised. CVST was diagnosed at presentation in over half of cases, although a proportion were identified later during admission following clinical deterioration [3]. Smaller cohort studies have reported higher rates of up to 5% in pneumococcal meningitis [6], suggesting that the true incidence may be underestimated, particularly in patients who do not undergo routine venous imaging.

Notably, in contrast to most reported cases where CVST is recognised at presentation or during clinical deterioration [3], the diagnosis in this case was made only after completion of a full 14-day course of antimicrobial therapy. This occurred in the context of persistent but non-specific symptoms and normalised inflammatory markers, without new focal neurological deficits. This delayed presentation highlights the potential for CVST to remain clinically occult even after apparent biochemical resolution of infection.

CVST in the context of meningitis is frequently associated with more severe disease, with patients more frequently developing focal neurological deficits [3]. Otitis media, mastoiditis, and sinusitis are commonly reported and may contribute to local spread of infection to adjacent venous structures [3]. The transverse sinus is the most frequently involved site, followed by the sigmoid and superior sagittal sinuses, with internal jugular vein involvement described in a minority of cases [3]. Multiple venous segments may be affected, and associated radiological findings can include cerebral infarction and, less commonly, intracerebral haemorrhage [3]. In this case, imaging demonstrated extensive thrombosis involving multiple contiguous venous territories, reflecting a more extensive pattern of venous involvement.

Management of CVST relies on prompt anticoagulation alongside treatment of the underlying cause. Multiple international guidelines (such as the United Kingdom, European, German, and the American Heart Association/American Stroke Association) [7-10] recommend that patients with suspected CVST undergo neuroimaging with CT or MRI including venography, followed by initiation of full-dose anticoagulation unless contraindicated. Anticoagulation is advised even in the presence of intracranial haemorrhage, as evidence suggests it is both safe and effective in preventing thrombus propagation and improving outcomes [7]. Low molecular weight heparin (LMWH) is generally preferred in the acute phase due to its more predictable pharmacokinetic profile and lower risk of complications compared to unfractionated heparin [7-10]. Following the acute phase, patients are typically transitioned to oral anticoagulation for a duration of three to twelve months depending on whether the event is provoked and the presence of ongoing risk factors [7-10].

In cases of infection-associated (septic) CVST, management should also include appropriate antimicrobial therapy. Current evidence suggests that the approach to anticoagulation does not differ significantly between septic and aseptic CVST [9]. In the present case, the patient had already completed an appropriate course of intravenous antibiotics at the time of CVST diagnosis. He was commenced on therapeutic-dose LMWH following multidisciplinary discussion with haematology, in keeping with guideline recommendations. Subsequent follow-up imaging demonstrated complete recanalisation, supporting the effectiveness of this approach in a provoked setting.

## Conclusions

CVST is a rare but important complication of pneumococcal meningitis that may be underrecognised, particularly in patients without overt neurological deterioration. This case highlights that CVST can present in a delayed manner, after completion of appropriate antimicrobial therapy and normalisation of inflammatory markers. Persistent headache or meningism should therefore prompt consideration of this diagnosis, including in those patients without new focal neurological deficits.

Early neuroimaging with venography is essential for diagnosis, and prompt initiation of anticoagulation remains the cornerstone of management. Favourable clinical and radiological outcomes can be achieved even in cases with extensive thrombus burden, as demonstrated in this case. Clinicians should maintain a high index of suspicion and pursue timely investigation and treatment, as early diagnosis and anticoagulation can improve outcomes.
